# Psychometric properties of Brief‐Balance Evaluation Systems Test (Brief‐BESTest) in evaluating balance performance in individuals with chronic stroke

**DOI:** 10.1002/brb3.649

**Published:** 2017-02-18

**Authors:** Meizhen Huang, Marco Y. C. Pang

**Affiliations:** ^1^ Department of Rehabilitation SciencesThe Hong Kong Polytechnic UniversityHong KongHong Kong

**Keywords:** balance, psychometrics, rehabilitation, stroke

## Abstract

**Objective:**

To examine the psychometric properties of the Brief‐Balance Evaluation Systems Test (Brief‐BESTest) in individuals with chronic stroke.

**Materials and Methods:**

This was an observational study with repeated measurements involving 50 participants with chronic stroke [mean (SD) age: 59.2 (7.3) years]. Each participant with stroke was evaluated with the Brief‐BESTest, Berg balance scale (BBS), Postural Assessment Scale for Stroke Patients (PASS), Fugl‐Meyer Motor Assessment (FMA), Chedoke‐McMaster Stroke Assessment (CMSA), Montreal Cognitive Assessment (MoCA), and Geriatric Depression Scale (GDS). Two raters (rater 1 and 2) provided the Brief‐BESTest scores of the first 27 participants independently to establish inter‐rater reliability. After 15 min of rest, the same 27 participants were evaluated with the Brief‐BESTest again by rater 1 to establish intra‐rater reliability. The Brief‐BESTest scores of the stroke group were also compared with those of the control group [*n *= 27, mean (SD) age: 56.7 (7.7) years].

**Results:**

The Brief‐BESTest had no substantial floor and ceiling effects, good intra‐rater (ICC_2,1_ = 0.974) and inter‐rater (ICC_2,1_ = 0.980) reliability and internal consistency (Cronbach's alpha = 0.818). The minimal detectable change at 95% confidence level was 2 points. The Brief‐BESTest showed moderate to very strong correlations with other balance (BBS and PASS) and motor impairment (FMA, CMSA) measures (*r*_s_ = .547–.911, *p *< .001), thus revealing good concurrent and convergent validity. Its correlation with measures that evaluated other constructs was weaker (MoCA:* r*_s_ = .437, *p *= .002) or non‐significant (GDS:* r*_s_  = −0.152, *p *= .292), thus showing good discriminant validity. Good known‐groups validity was established, as the Brief‐BESTest was effective in distinguishing participants with stroke from controls (cutoff score: <18, area under curve: 0.942), and individuals with stroke who required assistive device for their outdoor mobility from those who did not (cutoff score <14, area under curve: 0.810).

**Conclusions:**

The Brief‐BESTest has good reliability and validity in assessing balance function in individuals with chronic stroke.

## 1 Introduction

Balance dysfunction is common after stroke (Geurts, de Haart, van Nes, & Duysens, 2005), and is related to poorer mobility (Geiger, Allen, O'Keefe, & Hicks, 2001) and ability to perform activities of daily living (Hyndman & Ashburn, 2003), and falls (Quigley, 2016). Balance measures with good psychometric properties are crucial for accurate assessment of balance function.

Balance control involves many subsystems (Horak, 2006). However, common clinical balance measures such as the Functional Reach Test and Single Leg Stance, are single‐task measurements which could not assess multiple domains of balance and thus have limited value in directing treatment. The Berg Balance Scale (BBS) is a multi‐item generic balance measure, but its ceiling effects have been well documented when administered to individuals even as early as three months post‐stroke (Blum & Korner‐Bitensky, 2008; Mao, Hsueh, Tang, Sheu, & Hsieh, 2002). Postural Assessment Scale for Stroke Patients (PASS) is a stroke‐specific, multi‐item clinical assessment of balance (Benaim, Perennou, Villy, Rousseaux, & Pelissier, 1999). Similarly, its ceiling effect was apparent, with more than 75% of individuals achieving the highest possible PASS‐trunk control score at 90 and 180 days after stroke (Wang, Hsueh, Sheu, & Hsieh, 2005).

A more comprehensive balance assessment named the Balance Evaluation Systems Test (BESTest) was developed by Horak, Wrisley, and Frank (2009). It was designed to assess six subsystems of balance control (i.e., biomechanical constrains, stability limits/verticality, anticipatory postural responses, postural responses, sensory orientation, and stability in gait) (Horak et al., 2009). Despite its excellent psychometric properties in various populations (Chinsongkram et al., 2014; Horak et al., 2009; Jacobs & Kasser, 2012; Leddy, Crowner, & Earhart, 2011a), there are concerns with redundancy of items and long administration time involved (40–60 min) (Horak et al., 2009; Padgett, Jacobs, & Kasser, 2012). To address these limitations, the 14‐item Mini‐BESTest was developed (Franchignoni, Horak, Godi, Nardone, & Giordano, 2010). However, one limitation of the Mini‐BESTest is that it only assesses dynamic balance (Leddy, Crowner, & Earhart, 2011b; Padgett et al., 2012), with two of the six subsystems (i.e., “biomechanical constraints” and “stability limits/verticality”) in the original BESTest being omitted (Franchignoni et al., 2010). Thus, the Brief‐BESTest, which has been more recently developed, retains the theoretical basis of the original BESTest (Padgett et al., 2012). The eight test items cover all six balance subsystems and the administration time is less than 10 min, which makes it more feasible in daily clinical practice (Chan & Pang, 2015; Duncan et al., 2013; Leddy et al., 2011b; Padgett et al., 2012). Its reliability was comparable with the BESTest and Mini‐BESTest among individuals with Parkinson's disease (Leddy et al., 2011b), multiple sclerosis (Padgett et al., 2012), and total knee arthroplasty (Chan & Pang, 2015). However, the psychometric properties of Brief‐BESTest have not been evaluated among individuals with chronic stroke. It is essential to establish its reliability and validity before the Brief‐BESTest can be used in stroke research and clinical practice. The objective of this study was to examine the floor and ceiling effects, reliability, and validity of the Brief‐BESTest in individuals with chronic stroke.

## 2 Material and Methods

### 2.1 Study design

This was an observational study with repeated measurements. Floor and ceiling effects, internal consistency, intra‐rater and inter‐rater reliability, and concurrent validity (i.e., the measurement to be tested should have high correlations with a gold standard or criterion measure) (Portney & Watkins, 2009), convergent validity (i.e., the tool to be tested should have high correlation with measures that evaluate similar or related constructs) (Portney & Watkins, 2009), discriminant validity (i.e., the tool to be tested should have low correlation with measures that evaluate different attributes) (Portney & Watkins, 2009) and known‐groups validity (i.e., the measurement to be tested should be able to distinguish between individuals who are known to have the attribute being measured and those who are not) (Portney & Watkins, 2009) of the Brief‐BESTest were assessed in a group of individuals with stroke. To establish known‐groups validity, a control group was included to enable us to assess the difference in the Brief‐BESTest scores between the stroke group and control group. All the raters involved in this study were post‐graduate students in physiotherapy. The training involved reading the BESTest manual and watching an official demonstration video, followed by hands‐on practice. Pilot testing was done on two stroke patient volunteers (Chan & Pang, 2015). A specialist in neurological physiotherapy observed these pilot testing sessions to ensure that all raters performed the Brief‐BESTest and other assessments correctly before the collection of actual data .

### 2.2 Study participants

Individuals with stroke were recruited from a patient self‐help group during the period between September 2015 and January 2016 via convenience sampling. Inclusion criteria were: aged ≥18‐year, diagnosis of stroke for ≥6 months and community‐dwelling. Exclusion criteria included: history of neurological conditions other than stroke, inability to follow 2‐step commands, other severe medical conditions. Controls were recruited from the local community with the same eligibility criteria, except that there was no history of stroke. We did not set any minimum requirement for balance or mobility in our inclusion or exclusion criteria, because including individuals with a wide range of balance ability would provide us with a clear picture of the ceiling and floor effects of the Brief‐BESTest. Each potential participant was first screened by a telephone interview, followed by a face‐to‐face assessment session to ensure eligibility. Ethics approval was granted by the Human Research Ethics Subcommittee of the University. Written informed consent was obtained from all study paricipants.

### 2.3 Sample size estimation

The sample size estimation was based on a significance level of 0.05 and power of 0.8 using the Power Analysis and Sample Size Software Program (PASS 2005, NCSS, LLC, US). For inter‐rater and intra‐rater reliability analysis, an intraclass correlation coefficient (ICC) of 0.90 was assumed based on previous research on reliability of the Brief‐BESTest (Chan & Pang, 2015; Duncan et al., 2013; Padgett et al., 2012). With a null ICC at 0.75 (acceptable level of reliability), and expected ICC at 0.90, the sample size required would be 27 for the reliability analysis.

For concurrent and convergent validity, moderate to high correlations (*r *= .79) between the Mini‐BESTest and the BBS (*r *= .83) and Chedoke‐McMaster Stroke Assessment leg score (*r *= .53) and foot score (*r *= .64) among people with chronic stroke were identified by Tsang, Liao, Chung, & Pang (2013). Chan and Pang found a moderate correlation between the Brief‐BESTest and BBS (*r *= .74) among people with total knee arthroplasty (Chan & Pang, 2015). Thus, assuming a medium‐to‐large effect size (*r *= .4), a minimum sample size of 44 participants with stroke would be required.

For known‐groups validity, Padgett et al. (2012) showed that the Brief‐BESTest had good ability to differentiate between individuals with and without neurological disorders, yielding large effect sizes of 1.18–1.24. Assuming a large effect size (convention: *d *= 0.8), a minimum sample size of 26 individuals with stroke and 26 controls would be required to detect a significant between‐group difference in the Brief‐BESTest scores.

In summary, we aimed to recruit a minimum of 44 individuals with stroke and 26 control participants.

### 2.4 Measurement tools

As the aim of this study was to evaluate the psychometric properties of the Brief‐BESTest, the 8‐item Brief‐BESTest was the main measure of interest. Each individual item was rated on an ordinal scale of 0 to 3, yielding a maximal possible score of 24. Higher scores denote better balance performance (Padgett et al., 2012).

To establish concurrent validity, how well the Brief‐BESTest was correlated with other established balance measures should be assessed. Therefore, two other commonly used balance measures, namely, the Berg Balance Scale (BBS) and Postural Assessment Scale for Stroke Patients (PASS) were also included. The BBS contains 14 items, each of which was rated on an ordinal scale of 0 to 4 (maximum score: 56) (Godi et al., 2013; Mao et al., 2002). The BBS had good intra‐rater reliability (ICC = 0.98), inter‐rater reliability (ICC = 0.97) (Godi et al., 2013; Mao et al., 2002). On the other hand, the PASS consists of 12 items, and the score range for each item was from 0 to 3, yielding a maximum score of 36 (Benaim et al., 1999). The intra‐rater and inter‐rater reliability (0.84 and 0.99 respectively) and concurrent validity (correlation with BBS: 0.92–0.95) were good (Chien, Hu, Tang, Sheu, & Hsieh, 2007; Mao et al., 2002).

To establish convergent validity, the association between the Brief‐BESTest and measures that evaluate similar or related attributes should be examined. Balance ability should be closely related to motor recovery. Thus, two measures of motor recovery, namely, the Chedoke‐McMaster Stroke Assessment (CMSA‐leg and foot) and Fugl‐Meyer Motor Assessment‐Lower Extremity (FMA‐LE) were also administered. For CMSA‐leg and foot, motor recovery in the affected leg and foot was evaluated with a scale from 1 (no recovery) to 7 (full recovery) (Gowland et al., 1993). The intra‐rater and inter‐rater reliability for CMSA‐leg (ICC = 0.98 and 0.85), and CMSA‐foot (ICC = 0.94 and 0.96) was good (Gowland et al., 1993). For FMA‐LE, each item was scored on a 3‐point scale ranging from 0 to 2 (maximum score of 34) (Hiengkaew, Jitaree, & Chaiyawat, 2012). The FMA‐LE had good intra‐rater and inter‐rater reliability (0.95 and 0.92 respectively) among individuals with chronic stroke (Hiengkaew et al., 2012).

To assess the discriminant validity, the relationship between the Brief‐BESTest and measures that evaluated other traits should be assessed. Cognition and mood are two very important attributes that are distinct from balance measure. Hence, Montreal Cognitive Assessment (MoCA) and Geriatric Depression Scale‐Short Form (GDS) were also included. The MoCA assesses cognition, which could yield a total score ranging from 0 to 30. Higher scores are indicative of better cognitive ability (Wong et al., 2009). Its intra‐rater and inter‐rater reliability (0.96 and 0.87) was good (Wong et al., 2009). The 15‐item GDS was used to indicate the severity of depressive symptoms (score range: 0–15) (Mui, 1996). The test‐retest reliability was good (ICC = 0.75) in individuals with stroke (Mui, 1996).

### 2.5 Procedures

#### 2.5.1 Stroke group

Each participant with stroke underwent a single assessment session and relevant demographic data (e.g., age, medical history) were obtained through an interview conducted at the beginning of the session. All participants in the stroke group were evaluated with the Brief‐BESTest first, followed by GDS, MoCA, BBS, PASS, FMA‐LE, and CMSA‐leg and foot. Intermittent rest periods were given to minimize fatigue. The order of the tests was the same for all participants.

The first 27 individuals in the stroke group were invited to participate in the reliability testing. To establish inter‐rater reliability, rater 1 administered the Brief‐BESTest and provided the rating, while rater 2 observed the performance of the patient and provided the rating independently. For testing intra‐rater reliability, rater 1 repeated the same Brief‐BESTest on the same participants after a minimum of 15 min of rest. The typical duration of the assessment was 1.5 hr, including the rest periods. All assessment sessions were conducted in a university research laboratory.

#### 2.5.2 Control group

Participants in the control group underwent a single assessment session in the same university research laboratory. The Brief‐BESTest was administered only once by either rater 1 or 2. This was followed by the GDS, MoCA, and BBS. The order of the tests was the same for all participants in the control group. Stroke‐specific measurements, such as PASS, CMSA, and FMA‐LE were not administered. The typical duration of the assessment was 40 min, including the rest periods.

### 2.6 Data analysis

All statistical analyses were done by using SPSS version 21.0 (IBM Corporation, USA). The significance level was set at *p *≤ .05.

#### 2.6.1 Floor and ceiling effects

The skewness (γ_1_) of the Brief‐BESTest was assessed. A value of skewness greater than +1 indicates substantial floor effect while a value smaller than −1 indicates substantial ceiling effect (Chan & Pang, 2015). The proportion of participants obtaining the top 10% (i.e., total score >21) or bottom 10% (i.e., total score: <3) of the possible score range was also considered (Rodrigues Sde et al., 2013). The proportion of paticipants that is greater than 20% was considered as substantial ceiling or floor effects (Chan & Pang, 2015).

#### 2.6.2 Reliability

The internal consistency of the Brief‐BESTest was examined by Cronbach's alpha, based on the scores provided by rater 1 in the first trial. A value between 0.5 and 0.9 was considered as good internal consistency (Cortina, 1993). Intra‐class correlation coefficient (ICC_2,1_) was used for analyzing both the intra‐rater and inter‐rater reliability of the Brief‐BESTest total scores (<0.40: poor, 0.40 ≤ ICC≤0.75: adequate, >0.75: excellent) (Fleiss & Shrout, 1978). Wilcoxon test was used to compare the total scores between the two trials performed by rater 1. The intra‐rater and inter‐rater reliability of individual test items was examined by Kappa statistic (>0.8: almost perfect agreement, 0.61–0.8: substantial, 0.41–0.6: moderate, 0.21–0.4: fair, 0.01–0.2: slight, <0.01: poor) (Landis & Koch, 1977). Minimal detectable change at the 95% confidence level (MDC_95_) was calculated with the formula: MDC_95_  = 1.96 × SEM × √2 (Stratford & Goldsmith, 1997). Standard error of measurement (SEM) of the Brief‐BESTest total scores was calculated with the formula (Stratford & Goldsmith, 1997): SEM = SD × √(1‐ICC), where SD is the standard deviation of the Brief‐BESTest total scores and ICC is the reliability coefficient generated from the intra‐rater reliability analysis, based on data collected from the 27 individuals with stroke who participated in the reliability experiments.

#### 2.6.3 Validity

The Brief‐BESTest scores provided by rater 1 in the first trial were used for this analysis. Concurrent validity was assessed by correlating with other established balance measures (i.e., BBS, PASS). A high correlation was indicative of good concurrent validity. Convergent validity was examined by correlating with measurements that were supposedly related to balance function (i.e., CMSA‐leg and foot, FMA‐LE). A high correlation would denote good convergent validity. Discriminant validity was examined by correlating with measures that assessed different characteristics (i.e., GDS, MoCA). A low correlation would indicate good discriminant validity. Spearman's rho (*r*_s_) was used to examine the degree of association of Brief‐BESTest total scores with these measures (<0.2: very weak or no relationship, 0.2–0.4: weak, 0.4–0.6: moderate, 0.6–0.8: strong, and 0.8–1.0: very strong) (Portney & Watkins, 2009). To assess the known‐groups validity, the Brief‐BESTest total and item scores were compared between the stroke and control groups, and between users and non‐users of assistive device for their outdoor mobility within the stroke group, using Mann‐Whitney U tests. The receiver‐operating characteristics curve (ROC) analysis was used to identify the optimal cutoff score for differentiating between the stroke and control groups, and also between users and non‐users of assistive device within the stroke group. The area under curve (AUC), sensitivity and specificity values were generated by the ROC analysis. The AUC values were interpreted according to the guidelines described by Hosmer and Lemeshow (2000) (AUC ≥0.9: outstanding discrimination, AUC = 0.8–0.9: excellent discrimination; AUC = 0.7–0.8: acceptable discrimination). If the Brief‐BESTest had good known‐groups validity, there should be a significant difference in balance scores occurring between these groups. The AUC values would also be ≥0.7.

## 3 Results

Seventy‐seven individuals participated in the study (50 individuals with chronic stroke, 27 controls). The level of motor impairment in the affected lower extremity was moderate, as reflected by the FMA‐LE score (median = 19, IQR = 13–24). Thirty‐six individuals (72%) in the stroke group required an assistive device (e.g., cane, etc.) for their outdoor mobility (Table 1). None of the individuals used any assistive device during balance testing.

**Table 1:** Participant's characteristics^a^^,^ b

	Stroke (*n *= 50)	Control (*n *= 27)	*p*
Demographics			
Age, year	59.2 (7.3)	56.7 (7.7)	.164
Gender (male/female), *n*	32/18	11/16	.005^c^
Body mass index, kg/m^2^	24.4 (4.1)	25.9 (3.5)	.102
Geriatric Depression Scale (0–30)	3.5 (2–7)	3 (2–5)	.099
MoCA (0–30)	25 (21–28.25)	26 (24–27)	.972
No. of comorbidities per person, *n*	1 (0–2)	0 (0–1)	<.001^c^
No. of medications per person, *n*	2.5 (1–4)	0 (0–1)	<.001^c^
Stroke Characteristics			
Infract/Hemorrhage, *n*	30/20	—	
Post‐stroke duration, year	9 (3–13.5)	—	
Hemiplegic side (left/right), *n*	25/25	—	
CMSA			
Leg (1–7)	5 (4–6)	—	
Foot (1–7)	3 (1–4)	—	
FMA‐LE (0–34),	19 (13–24)	—	
Assistive device for outdoor walking			
None/Cane/quadripod/wheelchair/walking frame, *n*	14/25/4/6/1	27/0/0/0/0	
Balance performance			
Brief‐BESTest (0–24)	12.1 (5.2)	20.7 (1.7)	<.001^c^
Berg Balance Scale (0–56)	51 (48–55)	55 (55–56)	<.001^c^
PASS (0–36)	32.5 (30.8–34.0)	—	

The demographic and clinical characteristics of the stroke and control participants are shown.

a CMSA = Chedoke‐McMaster Stroke Assessment, FMA‐LE: Fugl‐Meyer Motor Assessment‐ Lower Extremity, IQR = Inter‐quantile Range, MoCA = Montreal Cognitive Assessment, PASS = Postural Assessment Scale for Stroke Patients.

b The results are expressed as mean (standard deviation) or median (first quartile‐third quartile).

c Significant difference between stroke group and control group (*p *≤ .05).

### 3.1 Floor and ceiling effects

**Figure 1:**
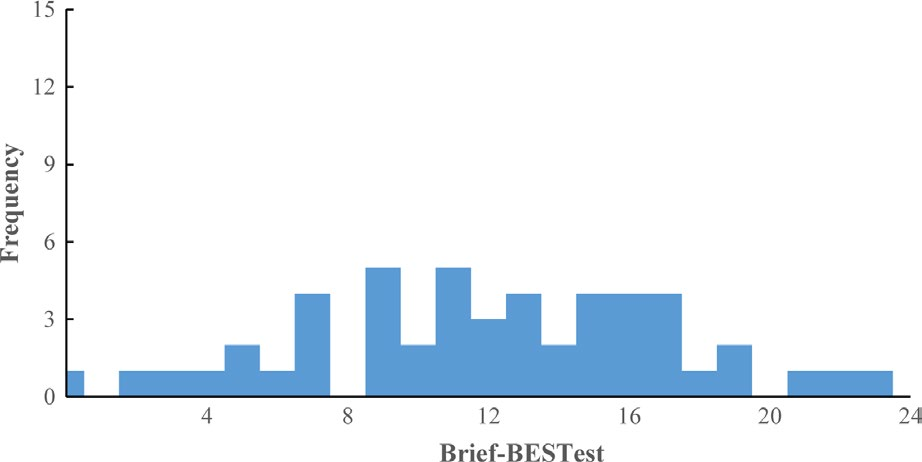
Score distribution of the Brief‐BESTest. The analysis was based on the data collected from 50 individuals with stroke. No ceiling or floor effect was identified

The distribution of the Brief‐BESTest scores within the stroke group is shown in Figure 1. The Brief‐BESTest scores showed no substantial skewness (γ_1_ = −0.139). The proportion of the participants in the stroke group who obtained the top 10% (i.e., total score >21) and bottom 10% (i.e., total score <3) of the possible score range of the Brief‐BESTest was only 0% and 4% respectively, indicating no substantial ceiling or floor effect.

### 3.2 Reliability

The Brief‐BESTest had good internal consistency (Cronbach's alpha = 0.818], intra‐rater reliability (ICC_2,1_ =  0.972, SEM = 0.823, *p *< .001) and inter‐rater reliability (ICC_2,1_ = 0.974, SEM =  0.772, *p *< .001) (Table 2). No significant difference was found between the scores generated from the two Brief‐BESTest trials conducted by rater 1 [mean (SD) trial 1: 13.8 (4.7), trial 2: 14.5 (5.1), *p *= .096], indicating no significant learning effect. The MDC_95_ value was 2. All items showed moderate to excellent intra‐rater and inter‐rater reliability except item 1 (hip/trunk lateral strength), which showed low inter‐rater agreement (Kappa = 0.304) and item 5 (compensatory stepping reaction on the paretic side), which showed low intra‐rater agreement (Kappa = 0.348) (Table 2).

**Table 2:** Intra‐rater and Inter‐rater reliability of Brief‐BESTest in individuals with stroke (*n *= 27)^a^

Brief‐BESTest item	Intra‐rater reliability										Inter‐rater reliability									
	Count^b^ (Test 1)				Count^b^ (Test 2)				Kappa	*p*	Count^b^ (Rater 1)				Count^b^ (Rater 2)				Kappa	*p*
	0	1	2	3	0	1	2	3			0	1	2	3	0	1	2	3		
1. Hip/trunk lateral strength	5	7	11	4	5	7	11	4	1.000	<.001^c^	5	1	10	11	5	7	11	4	0.304	.003^c^
2. Functional reach forward	0	4	22	1	0	4	23	0	0.870	<.001^c^	0	3	23	1	0	4	22	1	0.871	<.001^c^
3. Stand on one leg (paretic side)	17	2	6	2	17	1	6	3	0.864	<.001^c^	14	4	5	4	17	2	6	2	0.697	<.001^c^
4. Stand on one leg (non‐paretic side)	14	5	4	4	15	3	5	4	0.885	<.001^c^	13	6	5	3	14	5	4	4	0.833	<.001^c^
5. Compensatory stepping (paretic side)	3	11	4	9	3	8	10	6	0.348	<.001^c^	6	8	6	7	3	11	4	9	0.547	<.001^c^
6. Compensatory stepping (non‐paretic side)	3	9	6	9	3	6	6	12	0.586	<.001^c^	5	5	7	10	3	9	6	9	0.598	<.001^c^
7. Stand with eye closed on foam	1	0	1	25	1	0	1	25	1.000	<.001^c^	1	0	0	26	1	0	1	25	0.654	<.001^c^
8. Timed up and go	0	0	17	10	0	0	14	13	0.776	<.001^c^	0	0	17	10	0	0	17	10	1.000	<.001^c^

Brief‐BESTest total score	Test 1 Median (IQR)	Test 2 Median (IQR)	ICC, mean (95% CI)	*p*	Rater 1 Median (IQR)	Rater 2 Median (IQR)	ICC, mean (95% CI)	*p*
	14 (10–17)	14 (10–18)	0.972 (0.939, 0.987)	<.001^c^	19.5 (14.75–21)	14 (10–17)	0.974 (0.904, 0.990)	<.001^c^

The first 27 participants with stroke underwent the reliability testing. The Brief‐BESTest had good intra‐rater and inter‐rater reliability.

a Brief‐BESTest = Brief‐Balance Evaluation System Test; CI = confidence interval; ICC = intra‐class correlation coefficient; IQR = Inter‐quantile range.

b Count: the number of participants who received a score of 0, 1, 2 and 3 for each item in show.

c Reliability coefficient is statistically significant (*p *≤ .05).

### 3.3 Validity

The Brief‐BESTest total scores showed very strong correlations with the BBS (*r*_s_ = .872, *p *< .001) and PASS scores (*r*_s_ = .911, *p *< .001), thus showing good concurrent validity. It also yielded moderate to strong correlations with CMSA‐leg (*r*_s_ = .586, *p *< .001) and CMSA‐foot (*r*_s_ = .547 *p *< .001), and FMA‐LE (*r*_s_ = .664, *p *< .001), thus showing good convergent validity. Its correlation with MoCA was significant but weaker than the above measures (*r*_s_ = .437, *p *= .002), whereas its correlation with GDS was not statistically significant (*r*_s_ = −.152, *p *= .292), which was indicative of good discriminant validity of the Brief‐BESTest. There were significant differences in the Brief‐BESTest total scores and all individual item scores between the stroke and control groups (*p *< .001) (Table 3). The mean Brief‐BESTest total score among users of assistive device for their outdoor mobility was also significantly different from that among non‐users within the stroke group (*p *= .001). All item scores were significantly different between these two groups, except item 3 (standing on paretic leg: the participants were asked to lift the non‐paretic leg off of the ground without touching or resting the raised leg upon the other standing leg, and stay standing on the paretic leg as long as he/she could), item 4 (standing on non‐paretic leg: similar to item 3 described above but the participants were asked to lift the paretic leg off of the ground and stay standing on the non‐paretic leg as long as he/she could), and item 7 (standing on foam with eyes closed: the participants were required to stand on a foam, with both feet placed together, and maintain an upright standing posture for 30 s while keeping the eyes closed). The ROC analysis (Table 4) showed that the Brief‐BESTest total score was outstanding in discriminating between the stroke and control groups (cutoff: <18, AUC = 0.942), and excellent in identifying users of assistive device (cutoff: <14, AUC = 0.810).

**Table 3:** Known‐groups validity of Brief‐BESTest^a^

Test item	Stroke group (*n *= 50)					Control group (*n *= 27)					*p*	Users of assistive device within stroke group (*n *= 36)					Non‐users of assistive device within stroke group (*n *= 14)					*p*
	Count				Median (IQR)	Count				Median (IQR)		Count				Median (IQR)	Count				Median (IQR)	
	0	1	2	3		0	1	2	3			0	1	2	3		0	1	2	3		
Hip/trunk lateral strength	15	15	16	4	1 (0–2)	0	0	4	23	3 (3–3)	<.001^b^	14	14	7	1	1 (0–1)	1	1	9	3	2 (2–2.5)	<.001^b^
Functional reach forward	2	7	40	1	2 (2–2)	0	0	25	2	2 (2–2)	.012^b^	2	7	27	0	2 (1.3–2)	0	0	13	1	2 (2–2)	.018^b^
Stand on one leg (paretic side)	31	5	8	6	0 (0–2)	0	2	4	21	3 (3–3)	<.001^b^	25	2	4	5	0 (0–1.8)	6	3	4	1	1 (0–2)	.209
Stand on one leg (non‐paretic side)	29	9	4	8	0 (0–1.3)	0	2	4	21	3 (3–3)	<.001^b^	24	6	0	6	0 (0–1)	5	3	4	2	1 (0–2)	.069
Compensatory stepping (paretic side)	11	12	10	17	2 (1–3)	0	0	19	8	2 (2–3)	.034^b^	11	12	6	7	1 (0–2)	0	3	3	8	3 (1.8–3)	.002^b^
Compensatory stepping (non‐paretic side)	8	19	8	15	1 (1–3)	0	1	19	7	2 (2–3)	.011^b^	8	14	6	8	1 (1–2)	0	2	3	9	3 (2–3)	.001^b^
Stand with eyes closed on foam	5	11	5	29	3 (1–3)	0	0	2	25	3 (3–3)	.001^b^	4	9	3	20	3 (1–3)	1	2	2	9	3 (1.8–3)	.467
Timed up and go	5	0	32	13	2 (2–3)	0	0	0	27	3 (3–3)	<.001^b^	5	0	25	6	2 (2–2)	0	0	7	7	2.5 (2–3)	.010^b^

Brief‐BESTest total score	Median (IQR)	Median (IQR)	*p*	Median (IQR)	Median (IQR)	*p*
	14 (10–17)	21 (21–21)	<.001^b^	11 (7–13.8)	15.5 (13.8–19)	.001^b^

There was a significant difference in Brief‐BESTest total score between the stroke and control participants, and also between the users and non‐users of assistive device for outdoor mobility within the stroke group.

a Brief‐BESTest = Brief‐Best Evaluation System Test; IQR = Inter‐quantile range.

Count: the number of participants who received a score of 0, 1, 2, and 3 for each item is shown.

b Significant difference between stroke group and control group (*p *≤ .05), or between users and non‐users of assistive device within the stroke group.

**Table 4:** Receiver‐operating characteristics (ROC) analysis: known‐groups validity of Brief‐BESTest^a^

	AUC (95% CI)	Cutoff score	Sensitivity (95% CI)	Specificity (95% CI)
Discriminating individuals with stroke from controls	0.942 (0.888–0.996)	<18	0.880 (0.757–0.955)	0.926 (0.756–0.991)
Discriminating users of assistive device from non‐users within the stroke group	0.810 (0.684–0.935)	<14	0.750 (0.578–0.879)	0.786 (0.492–0.953)

The Brief‐BESTest was effective in discriminating the stroke participants from controls, as well as the individuals with stroke who required assistive device for outdoor mobility from non‐those who did not.

a AUC =  Area under curve; Brief‐BESTest = Brief‐Balance Evaluation Systems Test; CI = Confidence interval.

## 4 Discussion

This study showed that the Brief‐BESTest had no substantial floor and ceiling effects, excellent internal consistency, intra‐rater, and inter‐rater reliability when used in individuals with chronic stroke. Its concurrent, convergent, discriminant and known‐groups validity were also good.

### 4.1 Floor and ceiling effects

The Brief‐BESTest had no substantial ceiling or floor effect. As our participants were all ambulatory, many test items in the BBS (e.g., stand unsupported for 2 min; standing to sitting) and PASS (e.g., sitting without support for 5 min; supine to paretic side lateral) may not be challenging enough, leading to high BBS and PASS scores among our participants with stroke (Table 1). In contrast, the items in the Brief‐BESTest were generally more difficult for our participants. As shown in the item analysis, less than half of our participants were able to achieve the full item scores, with the exception of item 7 (stand with eyes closed on foam). Our results thus concurred with those found among individuals with total knee arthroplasty in that the Brief‐BESTest scores were less skewed than the BBS scores (*p *< .01) (Chan & Pang, 2015). Similar to our study, all individuals in their sample were ambulatory, which may explain the more pronounced ceiling effect of BBS. As much as 52.2% of their sample attained the full BBS score (i.e., 56 points) at 12 weeks post‐surgery, compared with only 8.7% for the Brief‐BESTest (Chan & Pang, 2015).

### 4.2 Reliability

The Brief‐BESTest had good internal consistency, indicating that the items were measuring the same underlying construct of balance. It also had good intra‐rater (ICC_2,1_ = 0.974) and inter‐rater (ICC_2,1_ = 0.972) reliability. Our findings were thus in line with those in individuals with total knee replacement (Cronbach's alpha = 0.97, inter‐rater ICC_2,1_ = 0.97 and intra‐rater ICC_2,1_ = 0.94) (Chan & Pang, 2015) and individuals with or without neurological problems (inter‐rater ICC_2,1_ =  0.94) (Padgett et al., 2012).

In our analysis of individual items, item 1 “hip/trunk lateral strength” in the Brief‐BESTest showed fair inter‐rater reliability only (Kappa = 0.304). The rating was partially based on the amount of force exerted by the rater's hands that provided support to the patients during testing. However, it was judged somewhat subjectively, and may vary among different raters. Rater 2 had to estimate the support given to the participants by rater 1 through mere observation, which may have caused the lower inter‐rater agreement. Item 5 “compensatory stepping on paretic side” also showed relatively low intra‐rater reliability (Kappa = 0.348). In this test, the participants were required to lean sideways against the rater's hands beyond the base of support. Two factors, namely, the extent of the lean and the amount of support provided by the rater's hands may vary across trials. The performance in compensatory stepping, particularly on the paretic side, may be substantially affected by even slight variations of these two factors.

### 4.3 Validity

The Brief‐BESTest had good concurrent validity, as revealed by its strong correlation with BBS and PASS. The results thus largely concurred with previous findings in older adults (O'Hoski, Sibley, Brooks, & Beauchamp, 2015), individuals with Parkinson's disease (Duncan et al., 2013) and total knee arthroplasty (Chan & Pang, 2015), where strong associations were found between the Brief‐BESTest and other established balance measures.

Convergent and discriminant validity of the Brief‐BESTest were also assessed. As expected, the Brief‐BESTest showed strong correlations with CMSA‐leg and foot and FMA‐LE scores, as the ability to maintain balance, to a large extent, requires the integrity of the motor system. Our findings thus were generally in line with those found in previous studies. For example, in individuals with total knee arthroplasty, there was a moderate correlation between the Brief‐BESTest and measures that assess constructs that were linked to balance such as the Functional Gait Assessment (*r*_s_ = .59–.72) (Chan & Pang, 2015). In the study by O'Hoski et al. (Portney & Watkins, 2009) involving a sample of 79 older adults (mean age: 68.7 years; age range: 50–87 years), the Brief‐BESTest was also moderately correlated with the Activities‐Specific Balance Confidence scale (*r *= .66) (O'Hoski et al., 2015).

In contrast, the Brief‐BESTest yielded a weaker correlation with MoCA, and no significant correlation with GDS, thus demonstrating good discriminant validity. GDS and MoCA measured very different traits (i.e., depression and cognition respectively) compared with the Brief‐BESTest, which may explain why the correlations between these measures and the Brief‐BESTest were weaker or even non‐significant. Yet, when compared with its correlation with GDS, the Brief‐BESTest had a stronger correlation with MoCA. It may be because relearning balance skills after stroke required a certain degree of cognitive ability. Indeed, Pahlman, Gutierrez‐Perez, Savborg, Knopp, and Tarkowski (2011) showed that patients with impaired cognition on admission and one year after stroke had significantly poorer balance performance than patients without cognitive impairments. In addition, only those individuals with intact cognitive function on admission and at the 1‐year follow‐up attained significant improvement in balance function after discharge. Their results thus highlighted the link between cognition and balance ability in individuals with stroke.

The Brief‐BESTest total scores and individual item scores demonstrated good known‐groups validity, as reflected by the significant difference between stroke and control group (*p *< .001), and the high AUC value (0.942). The Brief‐BESTest total scores were also useful in identifying those individuals with stroke who required an assistive device for outdoor mobility, but the discriminant accuracy was not as high (0.810). Item 3 (standing on paretic leg), item 4 (standing on non‐paretic leg), and item 7 (standing on foam with eyes closed) did not show a significant difference between users and non‐users of assistive device. Among these three items, the between‐group difference for item 4 might have reached statistical significance had a larger sample size been used (*p *= .069). Single‐leg‐standing on the paretic side (item 3) was very challenging for majority of individuals with stroke, regardless of whether they were users of assistive device or not. Indeed, a previous study found that this task has a severe floor effect in individuals with chronic stroke (Tsang et al., 2013). There was also a lack of significant between‐group difference for item 7 (standing on foam with eyes closed). Perhaps, the ability to use vestibular inputs for postural control may not be the most critical factor in determining the use of assistive device. Nevertheless, our results demonstrated that the Brief‐BESTest total score could reasonably differentiate users and non‐users of assistive device among individuals with stroke.

### 4.4 Study limitations

The findings can only be generalized to individuals with chronic stroke who are cognitively intact, self‐ambulatory, and community‐dwelling. All participants with stroke involved in this study were recruited from patient self‐help groups that organized regular physical and social activities for their members. These individuals may thus be more physically and socially active than their peers. The convenience sampling method used may have led to self‐selection bias. The evaluation of intra‐rater reliability was established by repeating the same measurements on the same day to minimize the need for the participants to travel to the laboratory twice within the same week. Ideally, the second test could be administered a few days after the first session. Nevertheless, our results showed that the learning effect was minimal. We only showed that the Brief‐BESTest can effectively discriminate stroke patients who used assistive device for their outdoor mobility from those who did not. A larger sample size will be required to further investigate the optimal cutoff score for discriminating individuals who used different types of assistive devices. Finally, the responsiveness of the Brief‐BESTest was not assessed. A prospective intervention study would be required to examine this issue.

### 4.5 Clinical implications

The Brief‐BESTest has good psychometric properties when administered to individuals with chronic stroke. The Brief‐BESTest thus provides a better option in assessing balance of this patient population, compared with the commonly used BBS. Another advantage is that it could assess all six balance subsystems, making it more useful for directing treatment than BBS or the Mini‐BESTest. From a practical point of view, the time required to administer the Brief‐BESTest is much shorter than the original BESTest and the BBS. The intra‐rater and inter‐rater reliability of the Brief‐BESTest is high among individuals with chronic stroke. This is important in many clinical settings where a number of clinicians may assess the same stroke patients at different times. The MDC_95_ value (2 points) established would also be useful for clinicians to determine whether the intervention has induced a real improvement in balance function in their patients, and for researchers to more accurately interpret the changes in Brief‐BESTest score in future studies of this field. The cutoff score of <14 may be useful in guiding the prescription of assistive device for individuals with stroke.

## 5 Conclusions

The Brief‐BESTest has good reliability and validity, and should be a useful tool in assessing the balance performance in individuals with chronic stroke in both clinical and research practice.

## Conflicts of Interest

The authors declare no conflict of interest.
